# Thinking While Moving or Moving While Thinking – Concepts of Motor-Cognitive Training for Cognitive Performance Enhancement

**DOI:** 10.3389/fnagi.2018.00228

**Published:** 2018-08-06

**Authors:** Fabian Herold, Dennis Hamacher, Lutz Schega, Notger G. Müller

**Affiliations:** ^1^Research Group Neuroprotection, German Center for Neurodegenerative Diseases (DZNE), Magdeburg, Germany; ^2^Department of Sport Science, Institute III, Otto von Guericke University Magdeburg, Magdeburg, Germany; ^3^Center for Behavioral Brain Sciences (CBBS), Magdeburg, Germany; ^4^Department of Neurology, Medical Faculty, Otto von Guericke University Magdeburg, Magdeburg, Germany

**Keywords:** exercise, cognition, dementia, dual task, cognitive enhancement

## Abstract

The demographic change in industrial countries, with increasingly sedentary lifestyles, has a negative impact on mental health. Normal and pathological aging leads to cognitive deficits. This development poses major challenges on national health systems. Therefore, it is necessary to develop efficient cognitive enhancement strategies. The combination of regular physical exercise with cognitive stimulation seems especially suited to increase an individual’s cognitive reserve, i.e., his/her resistance to degenerative processes of the brain. Here, we outline insufficiently explored fields in exercise-cognition research and provide a classification approach for different motor-cognitive training regimens. We suggest to classify motor-cognitive training in two categories, (I) *sequential motor-cognitive training* (the motor and cognitive training are conducted time separated) and (II) *simultaneous motor-cognitive training* (motor and cognitive training are conducted sequentially). In addition, simultaneous motor-cognitive training may be distinguished based on the specific characteristics of the cognitive task. If successfully solving the cognitive task is not a relevant prerequisite to complete the motor-cognitive task, we would consider this type of training as (IIa) *motor-cognitive training with additional cognitive task*. In contrast, in ecologically more valid (IIb) *motor cognitive training with incorporated cognitive task*, the cognitive tasks are a relevant prerequisite to solve the motor-cognitive task. We speculate that incorporating cognitive tasks into motor tasks, rather than separate training of mental and physical functions, is the most promising approach to efficiently enhance cognitive reserve. Further research investigating the influence of motor(-cognitive) exercises with different quantitative and qualitative characteristics on cognitive performance is urgently needed.

## Introduction

A crucial aspect of human living is motion. While the control of movements requires cognitive processes, moving probably influences cognition and its underlying processes (structures), too (Hamacher et al., 2015c). Cognition is a term covering a wide range of mental abilities that are necessary to percept, process, and interact with our environment (Bostrom and Sandberg, 2009; Borson, 2010). Therefore, intact cognitive processes are fundamental for human living. Across the life span, cognitive performance is influenced and changed by many factors. Normal aging is associated with a decline of cognitive functions such as processing speed and memory (Albert, 1997; Park et al., 2002; Buckner, 2004; Hedden and Gabrieli, 2004; Fjell and Walhovd, 2010). Moreover, old age is also a risk factor for developing neurological diseases like dementia (Fiest et al., 2016). Dementia has a negative impact on the cognitive performance of an individual and reduces the autonomy as well as the quality of life (Andersen et al., 2004; Scherder et al., 2011; Fiest et al., 2016). Remarkably, dementia is a major contributor to health care costs (Hurd et al., 2013; Wimo et al., 2013), and it is expected that the number of those affected by dementia will almost double every 20 years (Prince et al., 2013). Furthermore, the neuropathological signs of dementia aggravate with physical inactivity (Scherder et al., 2010). Physical inactivity and sedentary behavior are associated with reduced cognitive functions (Falck et al., 2016; Ku et al., 2017) and increased welfare costs (Janssen, 2012; Peeters et al., 2014), too. Unfortunately, the average time people are physically inactive in daily life has increased substantially in the last decades in western countries (Owen et al., 2010; Church et al., 2011). Taken together, the proportion of individuals with poorer cognitive capacities will increase in the next years, which makes it necessary to develop effective cognitive enhancement strategies (Colzato, 2016) that serve to enhance the cognitive reserve [defined as individual differences in how people process tasks; detailed description of term and concept could be found in Stern (2002, 2009)] and the resilience against neurodegeneration (Nithianantharajah and Hannan, 2009; Stern, 2012, 2013).

## Physical Activity and Cognition

Apart from a healthy diet and cognitive stimulation, (I) physical activity which is defined as any bodily, muscle-produced movement that increases the energy expenditure above ∼1.5 metabolic equivalent of task [1 MET = 1 kcal (4,184 kJ) × kg^-1^ × h^-1^] (Caspersen et al., 1985; Ainsworth et al., 2000; Mansoubi et al., 2015) and (II) physical exercise (training), which is defined as a planned, structured (repetitive) form of distinct physical activities (Caspersen et al., 1985; Howley, 2001; Budde et al., 2016) are proposed crucial for the preservation of cognitive functions and the enhancement of the cognitive reserve. For instance, physical activity and/or exercise were shown to improve cognition in children (Hillman and Schott, 2013; Chaddock-Heyman et al., 2014; Khan and Hillman, 2014; Verburgh et al., 2014; Ludyga et al., 2016), in adolescents (Verburgh et al., 2014; Esteban-Cornejo et al., 2015; Greeff et al., 2017; Li et al., 2017), in young and middle-aged adults (Verburgh et al., 2014; Cox et al., 2016), in older adults (Colcombe and Kramer, 2003; Bherer et al., 2013; Erickson et al., 2013; Carvalho et al., 2014; Northey et al., 2017), and in persons suffering from neurocognitive disorders such as dementia or mild cognitive impairment (Heyn et al., 2004; Smith et al., 2013; Groot et al., 2016; Ahn et al., 2017; Song et al., 2018). Moreover, regardless of whether physical exercise is conducted in a single (acute) exercise bout (Chang et al., 2012; Roig et al., 2013) and/or in form of multiple (chronic) exercise bouts (Bherer et al., 2013; Hötting and Röder, 2013; Roig et al., 2013; Esteban-Cornejo et al., 2015), it has been demonstrated to enhance cognitive performance. Furthermore, it is speculated that regular physical activity and physical exercising prevent cognitive decline and neurological diseases (Ahlskog, 2011; Sofi et al., 2011; Blondell et al., 2014; Paillard, 2015). However, which exact prerequisites (e.g., intensity, duration, frequency, type of exercise) make an exercise optimal for effectively enhancing cognition are largely unknown (Hillman et al., 2008; Lustig et al., 2009; Rolland et al., 2010; Bherer et al., 2013; Voelcker-Rehage and Niemann, 2013; Cai and Abrahamson, 2015; Esteban-Cornejo et al., 2015; Lauenroth et al., 2016; Batouli and Saba, 2017; Tait et al., 2017). Often, aerobic (cardiovascular) exercises like cycling, walking, or running are used in interventions to enhance cognitive fitness, especially in the elderly (Roig et al., 2013; Voelcker-Rehage and Niemann, 2013). For instance, a 6-month walking intervention leads to an increased hippocampal volume and improved memory performance in seniors (Erickson et al., 2011). Remarkably, preceding aerobic exercises boost performance in an alter reaction-time test (Pontifex et al., 2009) but not in executive functions when compared to preceding resistance exercises (Alves et al., 2012).

Interestingly, physical and motor fitness (coordinative abilities) are both related to cognitive performance in older individuals (Voelcker-Rehage et al., 2010) but do evoke differential structural adaptions (Voelcker-Rehage et al., 2011; Voelcker-Rehage and Niemann, 2013; Paillard, 2015) or correlate only with distinct cognitive functions (Marchetti et al., 2015). For instance, a 12-month aerobic exercise intervention led to increased activation in sensori-motor networks while coordinative training increased activation in the visual–spatial network during an executive function test (Voelcker-Rehage et al., 2011).

An exercise type that demands a high level of coordinative abilities is dancing (Dhami et al., 2014; Hamacher et al., 2015a,b). Participating in a 6-month dancing intervention enhanced attentional performance to a higher degree than participating in a fall prevention or Tai Chi Chuan program (Coubard et al., 2011). Additionally, in Parkinson disease dancing led to higher improvements in physical and cognitive functions than aerobic exercise (Hashimoto et al., 2015). Regular dancing has been associated with a lower risk of dementia (Verghese et al., 2003) presumably because it induces a larger increase in gray matter volume in frontal regions (Müller et al., 2016, 2017) and the hippocampus (Rehfeld et al., 2017).

Similar to chronic exercise, acute bouts of different exercise types have divergent effects on cognitive performance. Acute physical exercise with a high coordinative demand leads, in comparison to a purely aerobic exercise, to higher scores in attention tests (Budde et al., 2008), in working memory tests (Koutsandreou et al., 2016; Zach and Shalom, 2016), and in cognitive flexibility measures (Benzing et al., 2016). Taken together, acute and chronic physical exercises with high cognitive (coordinative) demands enhance cognitive performance. The beneficial effect of cognitively demanding exercises supports the recommendation of Pesce (2012) who proposes to shift the focus of exercise–cognition research from quantitative (e.g., exercise duration or intensity) to qualitative aspects (e.g., type of exercise).

Remarkably, it has been speculated that the combination of physical and cognitive exercise (also known as motor-cognitive training) could evoke a higher cognitive enhancement than cognitive or physical exercise alone (Kraft, 2012; Fissler et al., 2013; Bamidis et al., 2014; Lauenroth et al., 2016; Levin et al., 2017). The reasons why combined physical and cognitive exercise may be more effective than physical or cognitive training alone as well as the underlying neurophysiological mechanism are elucidated in the next section.

## Neurophysiological Mechanisms of Motor-Cognitive Training

To understand the beneficial effects of combined physical and cognitive exercises, it is advisable to consider our evolutionary past. From an evolutionary perspective, the human body and its organic systems exhibit a high adaptability to environmental constraints (Raichlen and Alexander, 2017). Moreover, over centuries the human physiology was adapted to an active lifestyle which ensured human survival, for example, through foraging, hunting, and fishing (Raichlen and Alexander, 2017). In industrialized nations, an increasing sedentary lifestyle (Owen et al., 2010; Church et al., 2011) probably promotes negative adaptations which minimize energy consumption, but also cause a decline of the motor and cognitive systems (Raichlen and Alexander, 2017). In contrast, the foraging activities (e.g., hunting) of our ancestors required a simultaneous use of cognitive and sensori-motor resources (e.g., walking and simultaneously scanning the environment for food) (Raichlen and Alexander, 2017). Such cognitive processes (e.g., detecting a deer in the landscape) were intertwined with related actions of the body (e.g., throwing a spear on a moving deer). The idea expressing that cognitive processes, bodily movements and interaction with the environment are interrelated is close to those theories known as “embodied cognition” and “embedded cognition” (Wilson, 2002; Pouw et al., 2014a,b). These considerations suggest that the combination of physical and cognitive challenges are essential to preserve or enhance the neural capacity which, in turn, ensures that cognitive processes function well (Kempermann et al., 2010; Fissler et al., 2013; Bamidis et al., 2014; Raichlen and Alexander, 2017).

**FIGURE 1:**
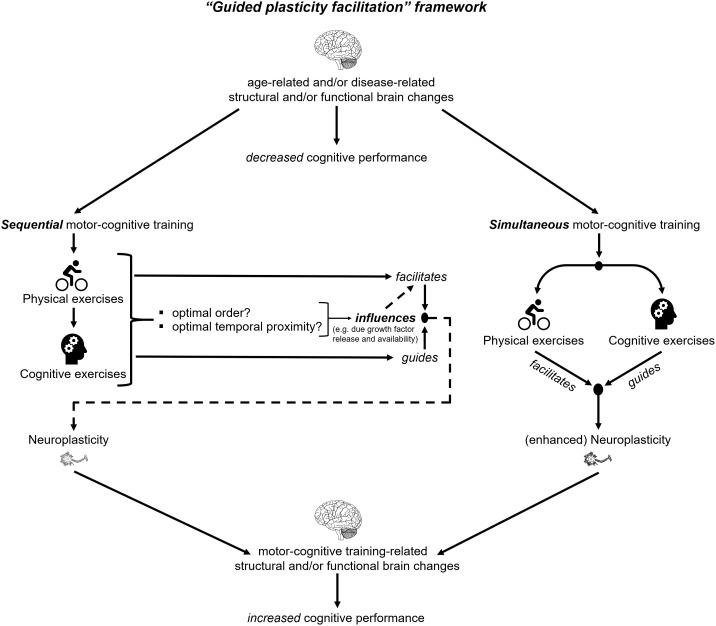
Schematic illustration of the “Guided plasticity facilitation” framework.

According to the “guided plasticity facilitation” framework (see **Figure 1**), the combination of physical and cognitive activities has positive synergistic effects that exceed the pure addition of the positive effects of cognitive and physical exercises (Kraft, 2012; Fissler et al., 2013). These super additive synergistic effects emerge from (I) the “facilitation effects” of physical exercises and (II) the “guidance effects” of cognitive exercises (see **Figure 1**). The “facilitation effect” of physical exercises triggers neurophysiological mechanisms, which promote neuroplasticity (Fissler et al., 2013). A possible physical exercise-induced mechanism, which promotes neuroplasticity is the enhanced release of neurotrophic factors such as the brain-derived neurotrophic factor (BDNF) (Knaepen et al., 2010; Coelho et al., 2013; Szuhany et al., 2015; Assis and Almondes, 2017; Dinoff et al., 2017). BDNF is associated with synaptogenesis and neurogenesis which may foster improved cognition (Cotman et al., 2007; Lu et al., 2013; Brigadski and Leßmann, 2014; Borror, 2017). Notably, an increased level of BDNF was observed during physical exercises and up to 60 min after cessation of the acute bout of physical exercises (Knaepen et al., 2010; Piepmeier and Etnier, 2015; Dinoff et al., 2017). Based on this observation, the “facilitation effects” of acute physical exercises seem transient and time-constrained (Fissler et al., 2013). While physical exercises induce neurophysiological processes that are fundamental for transient neuroplasticity (e.g., neurogenesis (Geibig et al., 2012), cognitive stimulation is assumed to “guide” these neuroplastic processes (Fissler et al., 2013; Bamidis et al., 2014). The “guidance effects” of cognitive exercises probably initiate distinct survival mechanisms of newborn cells (Fabel et al., 2009). These survival mechanisms are presumably part of a complex, multi-step mechanism that depends on the activation/stimulation of the newly generated synapses or neurons. The activation/stimulation of synapses and neurons occurs due to the execution of cognitive tasks and enables the functional integration of new neuronal structures in the respective brain circuits (Trachtenberg et al., 2002; Bruel-Jungerman et al., 2007; Bergami and Berninger, 2012; Bamidis et al., 2014). The integration in functional brain circuits seems to be crucial in order to stabilize the (by motor-cognitive training) induced neuroplastic changes. In addition to neuroplasticity, the stabilization of central nervous structures is likewise important to ensure good brain function (Kasai et al., 2003; Koleske, 2013).

## Types of Motor-Cognitive Training

**FIGURE 2:**
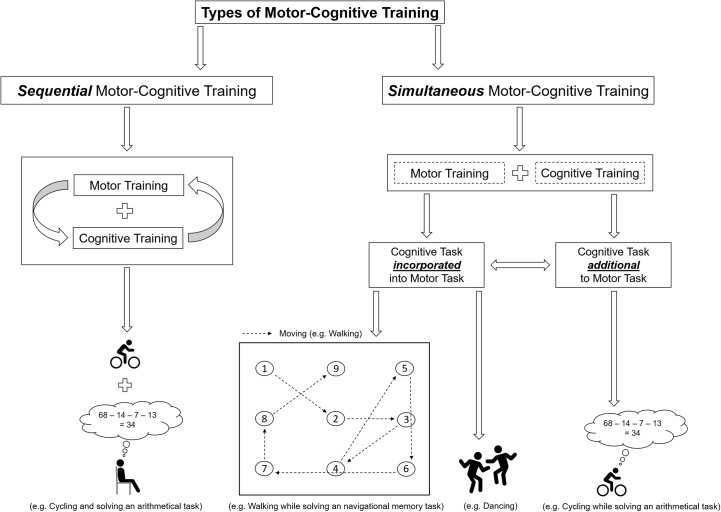
Schematic illustration of the classification of motor-cognitive training and a exemplifying illustrations of the differences between “additional” and “incorporated” cognitive training.

As shown in the framework in **Figure 2**, motor and cognitive exercises can be combined in several ways. In the first stage, we categorize motor and cognitive exercises based on their temporal order, which is a key factor for the effectiveness of the intervention (Roig et al., 2012; Fissler et al., 2013; Frith et al., 2017) in (I) sequential (or subsequent) motor-cognitive training and (II) simultaneous motor-cognitive training (see **Figure 2**).

### Sequential Motor-Cognitive Training

In sequential (or subsequent) motor-cognitive training, both the motor training and the cognitive training are each conducted at separate time points on the same day (prior to or after a bout of physical exercises) or at separate days (Tait et al., 2017). Commonly, the motor component of simultaneous motor-cognitive exercises compromises aerobic, resistance, balance, flexibility training, or a combination of them (for review see Lauenroth et al., 2016; Tait et al., 2017). In sequential motor-cognitive training interventions, the cognitive training was mainly focused on attention, memory, or multiple cognitive domains (for review see Lauenroth et al., 2016; Tait et al., 2017). An advantage of sequential motor-cognitive training (compared to simultaneous motor-cognitive training) is the absence of (1) possible dual-task costs [performance decrements in the motor task, the cognitive task, or in both tasks that may occur when two tasks are solved simultaneously (Hamacher et al., 2017)] and (2) prioritization effects (given a priority either to the motor task or the cognitive task), which vary individually and which might influence the results of the intervention. However, drawbacks of a sequential motor-cognitive training are the unknown appropriate load characteristics (frequency, length, duration, type of exercise, and the temporal order of the cognitive intervention and the motor intervention) for favorable cognitive outcomes (Lauenroth et al., 2016). For instance, the best benefit of an acute bout of motor exercises on cognitive performance was observed 11–20 min after cessation of the motor exercises (Chang et al., 2012). In contrast, another study reported that performing motor exercises 4 h after learning resulted in higher cognitive performance as compared to learning immediately afterward (van Dongen et al., 2016). Hence, it remains unclear whether cognitive training should be performed prior to or after motor exercises.

Interestingly, a recent review that compared the effects of sequential with simultaneous motor-cognitive training, reported that the simultaneous training significantly improved cognitive performance in various populations, whereas the sequential training yield inconclusive results (Tait et al., 2017). The latter finding may be explained by the fact that up to day it is unknown what time interval between motor and cognitive exercises is optimal (see **Figure 1**). Different time intervals may activate different neurobiological pathways (Tait et al., 2017). Based on these findings, simultaneous motor-cognitive training seems a more promising and time-efficient approach to foster cognitive functions than sequential training regimens.

### Simultaneous Motor-Cognitive Training

Simultaneous motor-cognitive training or motor-cognitive dual-task training is defined as training where both motor training and cognitive training are performed at the same time (Lauenroth et al., 2016). Simultaneous motor-cognitive training can be further classified regarding the supposed demands of the cognitive task (see **Figure 2**). We suggest to differentiate the two types of the supposed demands of cognitive tasks in simultaneous motor-cognitive training, which are presented in the following.

#### Motor-Cognitive Training With Additional Cognitive Tasks

A motor-cognitive training with additional cognitive task is similar to the “classical” dual-task approaches where, the secondary cognitive task is typically used as a *distractor* of the motor task (Schott, 2015). *Distractor* means that the additional cognitive task *is not a relevant prerequisite* to successfully complete the motor-cognitive task (*task nonrelevant*; e.g., walking and solving an arithmetic task or stationary cycling while citing alternate letters) and can be described as *Thinking while Moving.*

#### Motor-Cognitive Training With Incorporated Cognitive Tasks

When the cognitive task is “incorporated” into the motor task, the cognitive task *is a relevant prerequisite* to successfully solve the motor-cognitive task (e.g., walking to certain cones in a predefined order or dancing; see **Figure 2**) (Schott, 2015). Hence, this form of motor cognitive training can be described as *Moving while Thinking*.

We argue that incorporating the cognitive task(s) into the motor task(s) is more beneficial in terms of stabilizing neuroplasticity effects than using the cognition task as a distractor. Below, we will outline several reasons why this might be the case:

(1) Several studies suggest that the incorporation of a cognitive task into the motor task combines the advantageous effects of cognitive and motor training and leads to greater (motor-) cognitive improvements (Paas et al., 2003; Paas and Sweller, 2012; Moreau and Conway, 2013; Moreau, 2015; Ruiter et al., 2015). For example, it was observed that the integration of the cognitive task into physical activity enhanced cognitive learning in children more than performing physical activity unrelated to the cognitive task (Toumpaniari et al., 2015; Mavilidi et al., 2016, 2017, 2018).
(2) A training form that incorporates the cognitive task(s) into the motor task(s) is closer to daily life situations. For example, it is unlikely that an older person habitually solves an arithmetic task during walking, but it is likely that he/she walks through the supermarket while remembering what goods to buy and where to find those.
(2a) Exercise benefits are moderated by expectations (beliefs toward the effectiveness of the intervention) and preferences (Crum and Langer, 2007; Helfer et al., 2015). Given that even neurophysiological parameters are affected by individual preferences and expectations regarding the exercise intervention (Crabbe and Dishman, 2004; Schneider et al., 2009; Mothes et al., 2016), it can be assumed that the exercise-induced neural adaption processes and, in turn, the cognitive outcomes are influenced by those factors, too. For example, for an intervention to be effective, the adherence rate (compliance) to the training must be high. The latter is largely influenced by the subjectively experienced meaningfulness (perceived importance of the intervention with respect to individual situation) of the training (Trombly, 1995; Carlson et al., 2008; Fissler et al., 2013; Lautenschlager and Cox, 2013). As a cognitive task that is incorporated into a motor task closely resembles real-life situations, it is likely that such a combination is regarded as meaningful and, therefore, results in a high degree of adherence and in training success.
(2b) Older adults (Park et al., 2002) and persons with dementia (Pal et al., 2016), for instance, exhibit impaired visuospatial abilities (e.g., remembering the location of a certain product in the supermarket). As physical fitness effectively enhances cognitive functions (Bherer et al., 2013; Carvalho et al., 2014; Groot et al., 2016), an intervention, that targets the deficit in visuospatial memory, should, in our opinion, not only include a training of cognitive functions but also one of physical fitness. Moreover, given that the adaptions of the brain are task-specific (Dahlin et al., 2008; Green and Bavelier, 2012; Schlaffke et al., 2014), we propose that the ideal (motor-) cognitive training should also be specific (e.g., related to spatial memory) and should induce substantial transfer effects to everyday cognitive functions (e.g., remembering shopping list) as well as to activities of daily living (e.g., walking) (Jobe et al., 2001; Zelinski, 2009). These considerations suggest that the cognitive task should be incorporated into the motor task. In the context of our supermarket example remembering and walking to the respective goods can be combined. This approach is schematically depicted in **Figure 2**.
(3) If the cognitive task is incorporated into the motor task, no prioritization effects (which can be observed in motor-cognitive tasks with an additional cognitive task) would occur (Yogev-Seligmann et al., 2012a; Plummer et al., 2013, 2014; Plummer and Eskes, 2015). Such prioritization effects (giving priority either to the cognitive or the motor task) are known to influence motor and cognitive performance (Yogev-Seligmann et al., 2012b; Plummer et al., 2015a,b) as well as the activation of prefrontal structures (Lague-Beauvais et al., 2015). Theoretically, such prioritization-related effects could evoke distinct (undesired) adaptation processes.
(4) A further advantage of motor-cognitive training with incorporated cognitive tasks could be that multiple sensory systems are stimulated (due to the execution and control of the cognitive task and the motor task at the same point of time). Also, it is assumed that multisensory training environments better approximate all-day settings (which are ecologically more valid) and therefore provide an optimal basis for cognitive processes such as learning (Shams and Seitz, 2008).

Apart from a real-world setting as outlined for the proposed supermarket task, virtual reality environments offer the opportunity to combine cognitive and motor tasks without the need to construct a complex scenery (Tarr and Warren, 2002; Bruin et al., 2010). Respective approaches are the so-called “exergames” (Skjæret et al., 2016; Boissieu et al., 2017). Those video games, that are also a type of physical exercise task, can be classified within our proposed framework as simultaneous motor-cognitive training because they pose motor and cognitive demands simultaneously (Pichierri et al., 2012; Monteiro-Junior et al., 2016). However, as expectations always prove the rule, motor-cognitive tasks with an additional cognitive task are sometimes also common in a modern world [e.g., walking while texting or phoning (Hamacher et al., 2016)]. Nevertheless, we argue that this circumstance does not challenge our proposed framework; however, one would have to adapt the training characteristics if the aim is to improve the use of a smartphone while walking.

## Suggestions for Further Research Directions

We conclude that further exercise-cognition research is strongly needed, and we recommend that the major goals of this research should be:

(I) The identification of optimal qualitative (e.g., type of exercise) and quantitative training characteristics (e.g., frequency, duration, intensity, and temporal proximity of cognitive and motor training), which effectively and sustainably enhance the individual cognitive reserve and show transfer to daily life activities. Thereto, future research should consider the concept of “personalized training (or personalized medicine, if exercise is seen as a “medicament”), which aims to understand the heterogeneity in (motor-cognitive) exercise responsiveness (Buford and Pahor, 2012; Buford et al., 2013; Barha et al., 2017). “Personalized training” goes behind the “one-size-fits-all approach,” and the overall goal is to deliver tailored exercise prescriptions for the individual subject. Hence, participating subjects in future studies should be intensively screened and stratified into subgroups based on measures of physical fitness (e.g., cardiorespiratory fitness level or physical activity level assessed by questionnaires); motor fitness (e.g., coordinative abilities such as fine motor skills or hand/foot tapping speed); cognitive fitness (e.g., level of individual working memory performance); age, gender health status, and socioemotional status (e.g., measures of motivation, mood or stress). Furthermore, other potential moderators such as genotypic factors should be assessed if possible (Buford and Pahor, 2012; Barha et al., 2017). In order to achieve a “personalized training,” the programming of (motor-cognitive) exercise training should not solely be based on external training load characteristics (work performed by the subject, e.g., 70 % of maximal oxygen uptake) but also on internal training load characteristics (physiological and/or psychological effects in response to the performed task; e.g., individual level of blood lactate) (Bourdon et al., 2017; Burgess, 2017), because the same external training loads could lead to varying internal training loads (e.g., metabolic response such as blood lactate level) (Meyer et al., 1999; Scharhag-Rosenberger et al., 2010). For more detailed information on exercise prescriptions, we refer to the articles of Hofmann and Tschakert (2010), Mann et al. (2013), Bourdon et al. (2017), Burgess (2017), and Vanrenterghem et al. (2017).
(II) In order to prove whether the incorporation of cognitive tasks into acute or chronic exercise interventions (as described, for example, in **Figure 2**) is more effective regarding the enhancement of cognitive performance than (I) a sole motor training, (II) a sole cognitive training, (III) a sequential motor-cognitive training, or (IV) simultaneous motor-cognitive training with a non-task-relevant secondary cognitive task and in order to verify (or identify further) underlying, causal mechanisms, future work should elaborate on detailed modeling of neurobehavioral effects of (motor-cognitive) exercise interventions. Since physical exercises influence cognitive performance on multiple levels (Stillman et al., 2016) and also trough diverse short- and long-term mechanisms (Stimpson et al., 2018), different behavioral proxies (e.g., neuropsychological clinical tests and/or gait analysis), neuroimaging modalities (e.g., functional magnetic resonance imaging and/or functional near-infrared spectroscopy) as well as molecular and cellular measures (e.g., BDNF and/or lactate) should be used to validate such a neurobehavioral model.

## Author Contributions

FH and DH wrote the manuscript. LS and NM reviewed the drafted versions. All authors have read and approved the final version.

## Conflict of Interest Statement

The authors declare that the research was conducted in the absence of any commercial or financial relationships that could be construed as a potential conflict of interest.
